# Clinical and radiographic evaluation of Bio-Oss granules and Bio-Oss Collagen in the treatment of periodontal intrabony defects: a retrospective cohort study

**DOI:** 10.1590/1678-7757-2023-0268

**Published:** 2024-01-05

**Authors:** Jinmeng WANG, Wenjie CUI, Yang ZHAO, Lang LEI, Houxuan LI

**Affiliations:** 1 Nanjing University Affiliated Hospital of Medical School Department of Periodontology Nanjing China Nanjing University, Affiliated Hospital of Medical School, Nanjing Stomatological Hospital, Department of Periodontology, Nanjing, China.; 2 Nanjing University Affiliated Hospital of Medical School Department of Orthodontic Nanjing China Nanjing University, Affiliated Hospital of Medical School, Nanjing Stomatological Hospital, Department of Orthodontic, Nanjing, China.

**Keywords:** Intrabony defects, Periodontal regeneration, Bio-Oss, Bio-Oss Collagen

## Abstract

**Objective:**

This retrospective study aimed to analyze the clinical efficacy of two regenerative surgical methods — Bio-Oss granules combined with barrier membranes and Bio-Oss Collagen alone — and to help clinicians achieve better periodontal regeneration outcomes in the specific periodontal condition.

**Methodology:**

Patients who underwent periodontal regeneration surgery from January 2018 to April 2022 were retrospectively screened, and their clinical and radiographic outcomes at 6 months postoperatively were analyzed. The probing depth (PD), clinical attachment level (CAL), bleeding on probing (BOP), gingival recession (GR), distance from the cemento-enamel junction to the bottom of the bone defect (CEJ-BD), and depth of intrabony defects (INFRA) were recorded before the operation (T0) and 6 months after it (T1), and subsequently compared.

**Results:**

In total, 143 patients were included — 77 were placed in the Bio-Oss group and 66 were placed in the Bio-Oss Collagen group. All indicators, including PD and CAL at T1, showed significant differences compared to baseline, for both groups (P<0.001). PD reduction was greater in the group receiving the Bio-Oss Collagen treatment (P=0.042). Furthermore, in cases when the baseline PD range was 7-11 mm and the age range was 35-50 years, PD reduction was more significant for patients receiving the Bio-Oss Collagen treatment (P=0.031, 0.023). A linear regression analysis indicated that postoperative PD and CAL were positively correlated with baseline values, and that the efficacy tended to decrease with increasing age.

**Conclusion:**

Both the use of Bio-Oss Collagen alone and the use of Bio-Oss granules combined with barrier membranes resulted in significant effects in the treatment of periodontal intrabony defects. The Bio-Oss Collagen treatment generated more improvements in PD than the Bio-Oss granules combined with barrier membranes, particularly within the baseline PD range of 7-11 mm and the 35-50 years age group. Additionally, age was the main factor influencing the effectiveness of regenerative surgery for intrabony defects: older individuals exhibited fewer improvements.

## Introduction

Periodontal disease is a chronic, multifactorial, and infectious inflammatory disease associated with dental plaque biofilm.^1^ The removal of periodontal biofilm and eradication of periodontal inflammation is the current focus of the periodontal regimen in clinical practice. However, residual deep periodontal pockets associated with intrabony defects pose a significant challenge for clinical periodontists.^2^ Furthermore, long-term data have confirmed the association between residual probing pocket depth (PPD) and an increased risk of tooth loss.^3^ A pair of studies by Castro, et al.^4^ (2017) and Nibali, et al.^5^ (2020) recently reported that periodontal regenerative surgery is better than traditional periodontal flap surgery for treating intrabony defects. Kao, et al.^6^ (2015) pointed out that although there is variability in the results observed after periodontal regenerative surgery, overall, the clinical and/or histological outcomes of this method have been significantly better than those of traditional surgical approaches.

Previous studies on periodontal regenerative treatment methods have focused on two directions.^7^ One of them is the development of new surgical techniques. In recent years, minimally invasive surgical techniques (MIST)^8^ have been proposed for the treatment of intrabony defects, which have predictable long-term outcomes. The other direction involves the application of various regenerative materials.^9^ Three different concepts of regeneration are currently being investigated: barrier membranes, bone graft materials, and novel biomaterials and scaffolds, as well as their combinations. Guided tissue regeneration (GTR) has been successfully applied in the field of periodontal regeneration for many years. However, current GTR membranes lack specific periodontal regeneration properties, and the combination of GTR membranes with bone grafts is needed to enhance the regenerative effects of these methods on periodontal tissues.^10^

Bio-Oss and Bio-Oss Collagen are xenograft materials that are widely employed in dental practice. Xenograft materials have been successfully used in guided bone regeneration (GBR) and sinus floor elevation procedures.^9,11^ Bio-Oss is a natural, antigen-free, osteoconductive, bovine-derived, inorganic bone material with excellent biocompatibility and a low biodegradation rate, and it causes minimal tissue reaction.^12^ It is mainly composed of deproteinized bovine bone mineral (DBBM) particles, which are often used in combination with barrier membranes. Furthermore, its regenerative effect in the treatment of intrabony defects has been confirmed by multiple studies over several decades. Bio-Oss Collagen is composed of 90% Bio-Oss deproteinized DBBM particles and 10% biodegradable porcine collagen matrix. It combines the advantages of bone and collagen and provides excellent hemostatic properties.^13^ Bio-Oss Collagen has high porosity, which greatly increases its surface area and provides favorable scaffold conditions for bone formation.^14^ Whether used alone or in combination with collagen membranes, it can significantly improve the clinical efficacy of treatments for intrabony defects.^15^ Evidence from various studies indicates that Bio-Oss Collagen can be used for an ever wider range of surgical indications, such as alveolar ridge preservation (ARP) and socket augmentation, before or during implant placement.^16,17^

Although numerous studies have shown that both Bio-Oss granules and Bio-Oss Collagen have strong clinical effects in reducing probing depth (PD) and increasing clinical attachment level (CAL) in intrabony defects,^15,18,19^ it is unclear which of these resources is more effective. Few studies have compared both therapeutic methods in periodontal intrabony pockets: most studies comparing these resources focused on site preservation techniques.^20^ Although some studies have shown that bone grafting in combination with GTR can generate more predictable results,^21^ the clinical application of Bio-Oss with barrier membranes can be complex and may be associated with complications such as barrier membrane exposure, resulting in poor outcomes. Bio-Oss Collagen can be easily moistened with normal saline during clinical application and provide good stability of the surgical site, which makes it convenient for clinical manipulation.

According to existing literature, we hypothesize that Bio-Oss Collagen alone and Bio-Oss granules combined with barrier membranes will achieve comparable efficacy in periodontal regeneration. Therefore, the primary objective of this study is to determine the differences in clinical efficacy between the two regenerative methods in intrabony defects. The second objective is to explore the clinical efficacy of the methods in different periodontal conditions.

## Methodology

### Study design and patients

This retrospective study included 143 patients with periodontitis who underwent periodontal regenerative surgery for periodontal intrabony defects at the Nanjing Stomatological Hospital, Affiliated Hospital of Medical School, Nanjing University, between January 2018 and April 2022. After completing relevant etiological treatment, all patients underwent periodontal regenerative surgery. The efficacy of Bio-Oss granules combined with barrier membranes (n=77) in the treatment of intrabony defects was compared with that of Bio-Oss Collagen alone (n=66).

This study was approved by the Ethics Committee of the Affiliated Stomatological Hospital of Nanjing Medical University (Approval No. NJSH-2023NL-090). Patients provided written informed consent for the evaluation of retrospective clinical and radiographic data.

The inclusion criteria involved the following factors: 1) Stage III or IV periodontitis per 2018 criteria; 2) Ages 18-65 years; 3) Complete clinical and imaging data available for 6-month follow-up; 4) Clinical and radiographic confirmation of intrabony defects; 5) No systemic diseases or under control. The exclusion criteria involved: 1) Incomplete 6-month data; 2) Failing or refusing periodontal maintenance as scheduled; 3) Pregnancy during follow-up.

### Sample size

Based on prior studies,^18,22^ the CAL difference when using and not using Bio-Oss Collagen is 1.5 mm, with an expected standard deviation of 1.4 mm, a power of 0.90, and an α a set at 0.05. After calculations and considering a patient dropout rate of 20%, it was concluded that each group needed to be composed of 60 individuals.

### Clinical measurements

The clinical parameters before the surgery (T0) and 6 months after the surgery (T^1^) were recorded by an experienced periodontist who was blinded to the treatment, using a periodontal probe set at a probing pressure of 0.2 N. The recorded parameters included: (1) PD; (2) CAL; (3) bleeding on probing (BOP); (4) gingival recession (GR). The presence of BOP was recorded as 1 or 0.

### Radiographic measurements

The imaging data of intrabony defects were assessed as previously described.2^3^ The following parameters were recorded: (1) distance from the cemento-enamel junction to the most coronal extension of the inter-proximal bone crest (CEJ-BC); (2) distance from the cemento-enamel junction to the bottom of the bone defect (CEJ-BD); (3) depth of intrabony defects, defined as INFRA = (CEJ-BD) - (CEJ-BC). The radiographic data measurements were performed by the same periodontist who recorded the clinical data. The examiner underwent calibration training to ensure intra-examiner reproducibility. Each data set was measured three times by the examiner, achieving a standard deviation <0.5 mm for each parameter.

### Surgical procedures

All surgeries were performed by the same periodontist, who was not assisted by the physician performing the data measurements. A sulcular incision was made at the surgical site after administering local anesthesia. Ultrasonic and Gracey manual scalers were used to remove residual calculus and inflammatory granulation tissue. In the Bio-Oss group, Bio-Oss granules (Bio-Oss®; Geistlich Pharma AG, Wolhusen, Switzerland) soaked in normal saline were implanted into the intrabony defects, and pressure was applied evenly to restore the shape and match the surrounding alveolar bone. A suitably sized collagen membrane was selected for the defect area. In the post-debridement Bio-Oss Collagen group, Bio-Oss Collagen (Bio-Oss®; Geistlich Pharma AG, Wolhusen, Switzerland) was placed into the bone defect. Both groups’ incisions were sutured with 5-0 sutures. A superbond C&B bonding system (Sun Medical Co, Ltd. Moriyama, Japan) was used for patients with grade I or worse teeth mobility.

### Post-surgical period

The postoperative focus was on maintaining stability and infection control in the surgical area. Patients were required to rinse the oral cavity with 0.2% chlorhexidine twice a day for two weeks and were also administered 500 mg of amoxicillin three times a day for five days. Analgesics were given as needed for pain relief. The sutures were removed from the surgical site after two weeks. During the first four weeks after suture removal, patients were called to the hospital weekly to receive mild supragingival scaling and enhanced oral hygiene. Patients underwent periodontal support therapy in the 3rd and the 6th months after surgery, receiving additional supragingival scaling and reinforced oral hygiene guidance as needed. Clinical and imaging data were recorded 6 months after the surgery.

### Statistical analysis

For the statistical analysis, the SPSS 26.0 software was used. Statistical data were presented as mean ± standard deviation or percentage. A significance level of P=0.05 was used. A paired Student t-test was used to compare each group’s normally distributed baseline and postoperative parameters, while independent samples t-test was used for comparisons between the normally distributed parameters of the two groups. The Wilcoxon signed-rank test was used to compare each group’s non-normally distributed baseline and postoperative parameters, while comparisons between both groups’ non-normally distributed parameters were performed using the Mann-Whitney U test. The Chi-square test was used for comparing categorical variables. Linear regression analysis was used to assess the correlation between baseline and postoperative parameters. Stratified analyses based on different PD ranges and different age ranges were conducted to address confounding bias.

## Results

**Figure 1:**
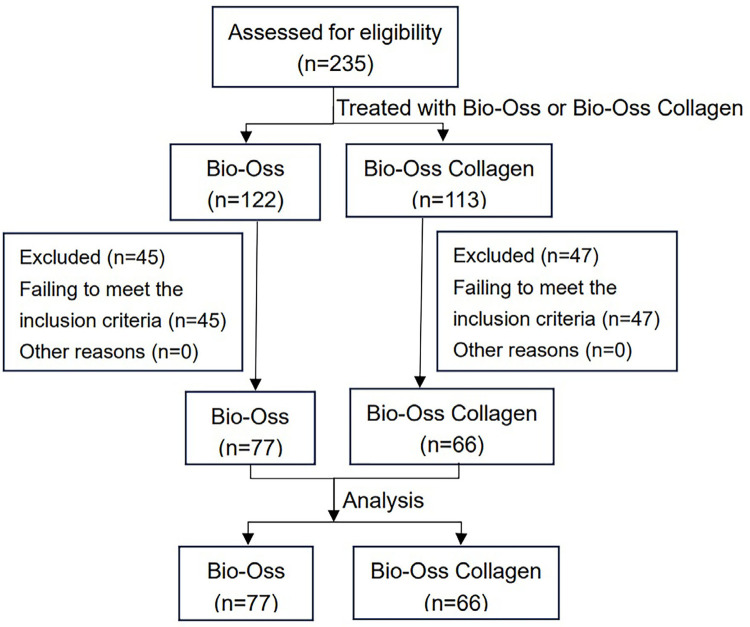
The flowchart of study design

In this study, 235 subjects’ data were extracted from the database. A total of 92 cases were excluded based on the inclusion criteria and 143 patients were included (Figure 1. The age range of the patients was 19-64. The Bio-Oss group (n=77) had an average age of 39.01±10.82 years (range 23-64), and the Bio-Oss Collagen group (n=66) had an average age of 39.79±10.44 years (range 19-63). The Bio-Oss group comprised 36 incisors/canines, 9 premolars, and 32 molars. The other group comprised 37 incisors/canines, 6 premolars, and 23 molars. The groups were followed up for an average of 5.49±0.64 months (Bio-Oss) and 5.73±0.83 months (Bio-Oss Collagen). No significant differences between both groups were found at baseline (P>0.05) (Table 1.

**Table 1:** Characteristics of research subjects at baseline (mean±SD or n/n%)

Variables	Bio-Oss	Bio-Oss Collagen	p value
Number (patients)	77	66	
Gender (male/female)	28/49	31/35	0.199
Age (years)	39.01±10.82	39.79±10.44	0.665
Tooth type			0.536
Incisors, canines	36 (46.75%)	37 (56.06%)	
Premolars	9 (11.69%)	6 (9.09%)	
Molars	32 (41.56%)	23 (34.85%)	
Follow-up time (months)	5.49±0.64	5.73±0.83	0.06

Table 2shows all the clinical and radiographic indicators at baseline and at month 6. There were no significant differences between both groups’ baseline parameters (P>0.05). When comparing the baseline and 6-month indicators, it was found that most changes were statistically significant and most indicators had P values < 0.001, except GR in the Bio-Oss Collagen group (P=0.049). At month 6, indicators in both groups showed improvement, except GR, which increased. PD and CAL were chosen as two key efficacy indicators and are presented in Figure 2 [Fig f03] as box plots with distributions correlating to pre- and post-surgery. Combining Table 2with Figure 2 [Fig f03], it can be observed that the mean baseline PD values of both groups were similar, but those in the Bio-Oss Collagen group had a more dispersed distribution, while the postoperative PD values in the Bio-Oss Collagen group were lower and more concentrated. The CAL indicator showed the opposite trend.

**Table 2:** Clinical and radiographic parameters at baseline and T1 (mean±SD or n/n%)

Variables	Bio-Oss (n=77)		Bio-Oss Collagen group (n=66)				
	T0	T1	T0	T1	p0	p^**b**^	p^**bc**^
PD (mm)	7.93±1.13	4.14±1.17	7.99±1.34	3.80±0.92	0.758	<0.001*	<0.001*
CAL (mm)	9.11±1.35	5.49±1.43	9.07±1.68	5.48±1.56	0.848	<0.001*	<0.001*
GR (mm)	1.10±1.02	1.31±1.21	1.17±1.05	1.65±1.21	0.689	0.049*	<0.001*
BOP (%)	77(100%)	31(40.26%)	66(100%)	27(40.91%)	--	<0.001*	<0.001*
CEJ-BD (mm)	6.98±2.28	3.82±1.79	7.01±1.82	3.69±1.85	0.949	<0.001*	<0.001*
INFRA (mm)	3.80±1.51	0.76±0.65	4.21±1.62	1.35±1.35	0.12	<0.001*	<0.001*

*Statistically significant p<0.05; T0: pre-operation; T1: 6 months after surgery. p0: comparison between T0 in the two group; pb: comparison between T0 and T1 in the Bio-Oss group; pbc: comparison between T0 and T1 in the Bio-Oss Collagen group. PD, probing depth; CAL, clinical attachment level; GR, gingival recession; BOP, bleeding on probing; CEJ-BD, the distance from the CEJ to the bottom of the bone defect; INFRA, the depth of intrabony defects

**Figure 2(a):**
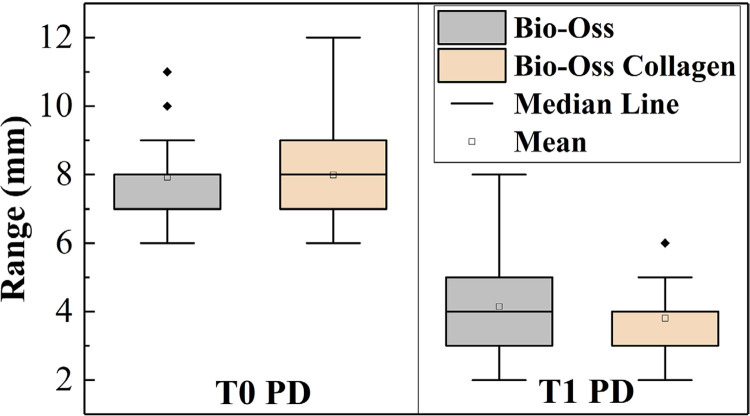
Box plot distribution of PD pre- and post-surgery

**Figure 2(b) f03:**
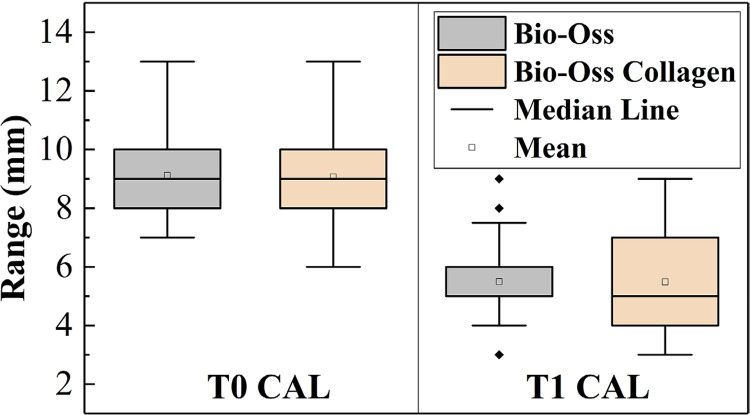
Box plot distribution of CAL pre- and post-surgery

Table 3shows the changes in various indicators. In the Bio-Oss group, ΔPD and ΔCAL values were 3.78±1.23mm and 3.62±1.24mm, respectively, while in the Bio-Oss Collagen group, these values were 4.20±1.11mm and 3.58±1.27mm, respectively. There was a significant difference in PD reduction between both groups: the Bio-Oss Collagen group showed a more significant improvement in PD (P=0.042). Other parameters did not show significant changes.

**Table 3:** Changes in parameters over the 6 month period after treatment (mean ± SD)

Variables	Bio-Oss (n=77)	Bio-Oss Collagen (n=66)	p value
ΔPD (mm)	3.78±1.23	4.20±1.11	0.042*
ΔCAL (mm)	3.62±1.24	3.58±1.27	0.849
ΔGR (mm)	-0.21±0.94	-0.48±0.73	0.06
DBOP (%)	46(59.74%)	39(59.09%)	0.937
DCEJ-BD (mm)	3.16±1.57	3.31±1.37	0.532
ΔINFRA (mm)	3.04±1.43	2.86±1.43	0.465

The histograms in Figure 3 [Fig f05] indicate that 97.0% of the ΔPD in the Bio-Oss Collagen group was distributed within the 3-7 mm range, while in the Bio-Oss group, 83.1% of the ΔPD was within 3-7 mm, and 15.6% was < 3 mm. Similarly, 97.0% of ΔCAL in the Bio-Oss Collagen group was distributed within the 3-7 mm range, while in the Bio-Oss group, 81.8% was within 3-7 mm, and 18.2% was < 3mm.

**Figure 3(a):**
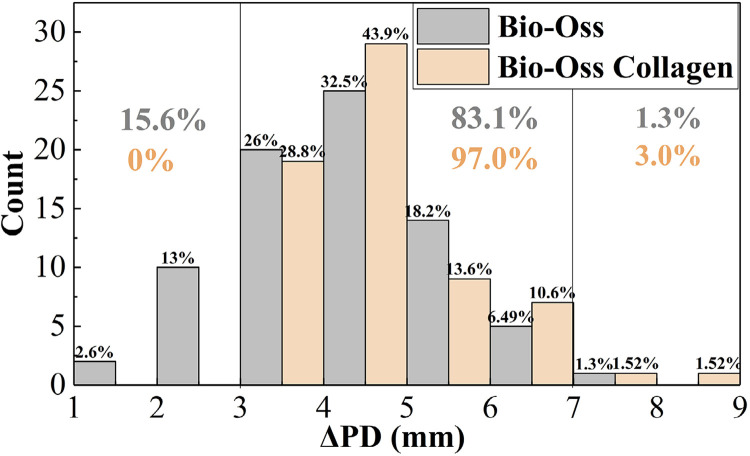
Histogram distribution of ΔPD pre- and post-surgery

**Figure 3(b) f05:**
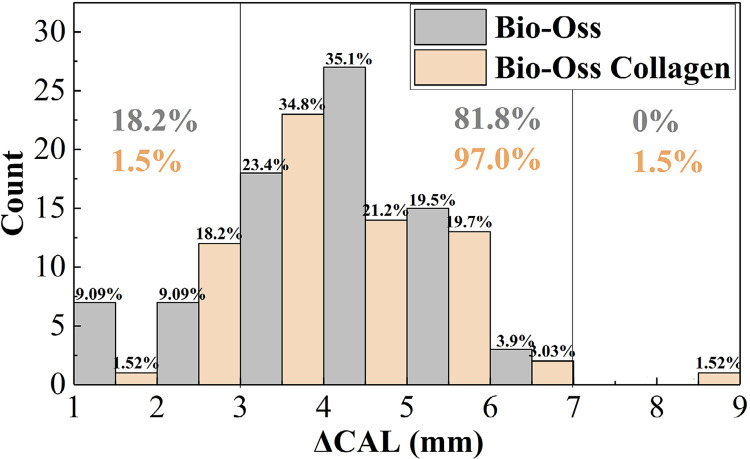
Histogram distribution of ΔPD pre- and post-surgery

Table 4shows that there was a more significant difference between the groups’ ΔPD (P=0.031) when the baseline PD was within the 7-11 mm range. The Bio-Oss Collagen group had a greater ΔPD, which indicates that it was more effective in this range. Table 5shows that there was a significant difference between the groups’ ΔPD (P=0.023) when patients were aged between 35 and 50 years. The Bio-Oss Collagen group had a greater ΔPD, which indicates that it was more effective in this age group. Figure 4 [Fig f07] presents the regression trends of PD and CAL before and after the surgery. This indicates that postoperative PD and CAL values have a positive linear correlation with the baseline values. Furthermore, Figure 4 shows that patients with PD and CAL values above the regression line had an average age of 41 years (41.33±10.99 and 41.02±10.69), while those with PD and CAL values below the regression line had an average age of 38 years (38.07±10.22 and 37.96±10.41).

**Table 4:** ΔPD (mm) in different baseline PD ranges

Severity	Bio-Oss (n=77)	Bio-Oss Collagen (n=66)	p value
PD<7mm	3.42±0.66	3.38±0.52	0.897
7mm≤PD<11mm	3.77±1.23	4.23±1.10	0.031*
PD≥11mm	5.00±1.73	5.67±0.58	0.561

**Table 5:** ΔPD (mm) in different age ranges

	Bio-Oss (n=77)	Bio-Oss Collagen (n=66)	p value
Age≤35	3.92±1.28	4.48±1.34	0.126
35<Age≤50	3.67±1.28	4.33±0.93	0.023*
Age>50	3.75±0.94	3.35±0.47	0.183

**Figure 4(b):**
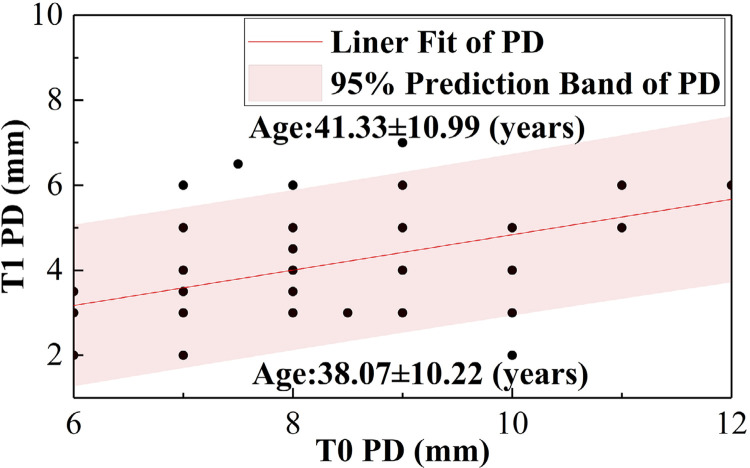
Linear regression trends of CAL pre- and post-surgery

**Figure 4(a) f07:**
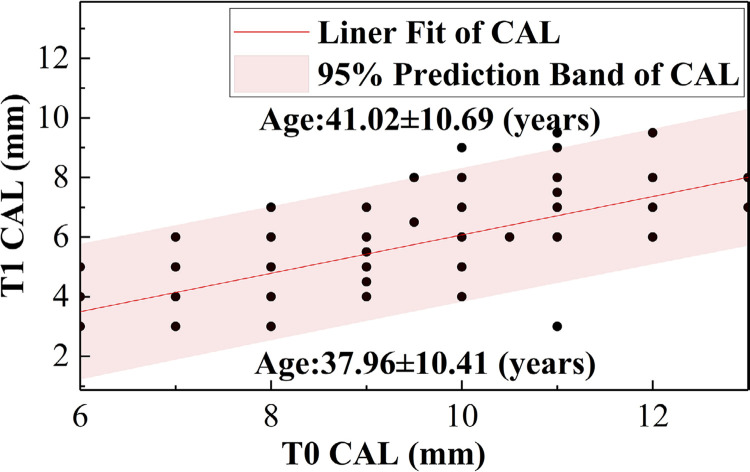
Linear regression trends of PD pre- and post-surgery

## Discussion

The primary objective of this retrospective study was to determine the differences in clinical efficacy between two regenerative surgical methods for periodontal intrabony defects. Some separate studies on these two methods have been conducted. A study carried out in 2003 histologically confirmed that using Bio-Oss Collagen alone in intrabony defects can promote new attachment formation in the periodontium.^24^ Subsequently, Hartman, et al.^15^ (2004) demonstrated that using only Bio-Oss Collagen for regenerating intrabony defects can significantly improve clinical indicators such as PD and CAL (6.8 mm and 5.3 mm, respectively) at 6 months postoperatively. The combined use of Bio-Oss and barrier membranes has proven effective for treating intrabony defects.^25^ Liu, et al.^25^ (2022) observed an average gain of 2.00 mm in CAL and an average bone increment of 3.00 mm one year after performing minimally invasive periodontal surgery. The available literature suggests that both regenerative surgical approaches generated significant therapeutic effects in the treatment of intrabony defects, which is in line with the findings of our study. This study also indicates that there is no significant difference in overall efficacy between these two methods; however, this efficacy may be influenced by some factors.

Studies have indicated that factors such as smoking^26^ and defect morphology^27^ impact treatment outcomes. This study assessed post-regeneration efficacy in various PD levels and age groups. The findings showed that the Bio-Oss Collagen treatment generated more improvement in the baseline PD range of 7-11 mm, which indicates that the suitable treatment method can be chosen based on specific periodontal conditions. Additionally, the two treatments exhibited similar effectiveness in both age groups, particularly in patients aged > 50 years, which suggests that minimally invasive approaches like Bio-Oss Collagen may be preferable for older patients. The results of the linear regression revealed that the post-operative improvement decreased with increasing age, implying that postoperative healing is worse in older patients.

When Bio-Oss Collagen is used in intrabony defects, the surgical area only needs to extend as far as the crest of the alveolar ridge or a smaller area. MIST has recently become a hot topic in many studies.^8,25^ The interest in Bio-Oss Collagen use in MIST has grown. Unlike Bio-Oss granules, Bio-Oss Collagen contains 10% porcine-derived collagen, which makes it easy to shape during surgery after blood infiltration.^28^ A study by Jung, et al.^29^ (2021) showed that after 5 months of GBR in the bone defect area of the implant site, Bio-Oss Collagen showed a more stable volume than Bio-Oss granules.

On the contrary, Bio-Oss granules comes in a dispersed granular form and is often used in conjunction with barrier membranes that cover the surgical site and prevent leakage of bone particles.^20^ Additionally, there may be adverse events such as early exposure of the barrier membrane, which significantly reduces the osteogenic effect.^30^ In this regard, the surgical scope of using Bio-Oss granules is bound to be larger than that of using Bio-Oss Collagen alone, resulting in greater surgical trauma for patients. Besides the difference in surgical scope, the cost and complexity of the procedures should also be considered. The combined use of Bio-Oss granules and barrier membranes is much more expensive compared to using Bio-Oss Collagen alone. Moreover, Bio-Oss Collagen is less complex to use, making it a better choice for young doctors. Therefore, when clinicians attempt to preserve natural teeth, they should consider multiple factors in conjunction to select the optimal treatment plan for patients.

The advantages of this study are the large sample size and the analysis of various clinical and radiological indicators. Additionally, when analyzing the characteristics of Bio-Oss Collagen, clinicians were provided with insights for improvement in MIST. However, there are still some limitations, such as the lack of consideration for factors such as smoking as well as types and angles of intrabony defects. Additionally, the allocation of patients was not according to the randomization process, which is also one of the limitations of the study. Furthermore, this study is retrospective, and the conclusions drawn need to be interpreted cautiously. Therefore, further randomized clinical trials with longer observation periods are still needed to validate our findings.

## Conclusions

In summary, the hypothesis was rejected. Regarding PD, Bio-Oss Collagen treatment demonstrated more improvements than Bio-Oss granules combined with barrier membranes, particularly within the baseline PD range of 7-11 mm and the 35-50 age group. Additionally, age was the main factor influencing the effectiveness of regenerative surgery for intrabony defects, with older individuals exhibiting fewer improvements.
